# Response of the carotid artery longitudinal motion to submaximal physical activity in healthy humans—Marked changes already at low workload

**DOI:** 10.14814/phy2.15580

**Published:** 2023-01-26

**Authors:** Åsa Rydén Ahlgren, Tobias Erlöv, Magnus Cinthio

**Affiliations:** ^1^ Department of Translational Medicine Lund University Lund Sweden; ^2^ Department of Medical Imaging and Physiology, Skåne University Hospital Lund University Malmö Sweden; ^3^ Department of Biomedical Engineering Lund University Lund Sweden

## Abstract

The longitudinal motion of the arterial wall, that is, the displacement of the arterial wall *along* the artery, parallel to blood flow, is still largely unexplored. The magnitude and nature of putative changes in longitudinal motion of the arterial wall in response to physical activity in humans remain unknown. The aim of this study was therefore to study the longitudinal motion of the carotid artery wall during physical activity in healthy humans. Using in‐house developed non‐invasive ultrasonic methods, the longitudinal motion of the intima‐media complex and the diameter changes of the right common carotid artery (CCA) in 40 healthy volunteers (20 volunteers aged 22–35 years; 20 volunteers aged 55–68 years) were assessed at rest and during submaximal supine bicycle exercise. In a subset of the subjects (*n* = 18) also intramural shear strain were analyzed. The longitudinal motion of the intima‐media complex underwent marked changes in response to physical activity, already at low workload; with most evident a marked increase of the first antegrade displacement (*p* < 0.001) in early systole. Likewise, the corresponding shear strain also increased significantly (*p* = 0.004). The increase in longitudinal motion showed significant correlation to increase in blood pressure, but not to blood flow velocity or wall shear stress. In conclusion, physical activity markedly influences the longitudinal motion of the carotid artery wall in healthy humans already at low load. A possible “cushioning” function as well as possible implications for the function of the vasa vasorum, endothelium, and smooth muscle cells and extracellular matrix of the media, are discussed.

## Graphical abstract

The longitudinal motion of the arterial wall, that is, the displacement of the arterial wall along the artery, parallel to blood flow, is still largely unexplored. Using in‐house developed non‐invasive ultrasonic methods we show, for the first time, that physical activity markedly influences the longitudinal motion of the carotid artery wall in healthy humans already at low load. Possible implications for cardiovascular function are discussed.

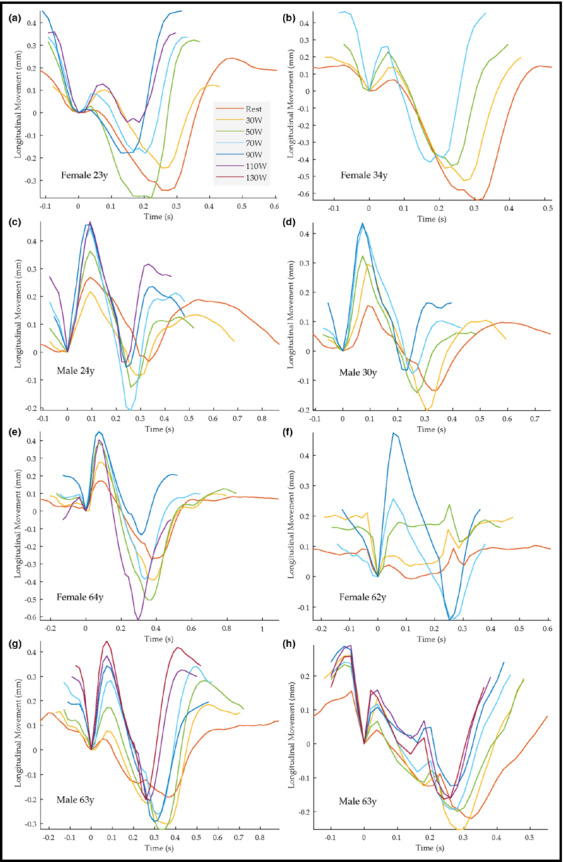

## 1 INTRODUCTION

Until about 20 years ago the longitudinal motion of the arterial wall has been assumed to be negligible in comparison with the diameter change (Nichols et al., 2005). We and others have, however, shown that the arterial wall of large arteries at rest do not only show a well‐known diameter change during the cardiac cycle, but also shows a distinct longitudinal displacement, that is, a motion along the artery parallel to blood flow, of the same magnitude as the diameter change (Ahlgren, Cinthio, Persson et al., 2012; Cinthio et al., 2006; Persson et al., 2002, 2003; Zahnd et al., 2011). We have also shown that the intima‐media complex, that is, the inner layers of the arterial wall, show a larger longitudinal motion than the adventitial region, indicating shear strain, and thus shear stress, within the arterial wall (Cinthio et al., 2006). Although most studies have been performed on the carotid artery and there are very few studies on other arteries, this seems to be general phenomena in both large elastic and predominantly muscular arteries (Cinthio et al., 2006). The function of this longitudinal motion, or displacement, is at present largely unknown. In studies on the carotid arteries, we and others have shown that healthy subjects can show dramatically different pattern of longitudinal motion of the intima media complex (Ahlgren, Cinthio, Persson et al., 2012; Cinthio et al., 2006; Rizi et al., 2020; Yli‐Ollila et al., 2013), and that these patterns of longitudinal motion are stable over months (Ahlgren, Cinthio, Persson et al., 2012) but undergo marked changes with aging (Cinthio et al., 2018).

So far, studies on the longitudinal motion of the arterial wall have mainly focused on the longitudinal displacement as a potential new marker of cardiovascular risk (Au et al., 2017; Svedlund & Gan, 2011; Svedlund et al., 2011; Taivainen et al., 2017, 2018; Tat et al., 2017; Zahnd et al., 2012). There has been less interest in the putative functions of the longitudinal motion for the normal performance of the cardiovascular system. In pharmacological experiments on the muscular porcine carotid artery, we, however, have shown that the longitudinal motion and resulting intramural shear strain undergo profound changes in response to our important circulatory hormones adrenalin and noradrenalin, that is, the catecholamines (Ahlgren, Cinthio, Steen et al., 2012), indicating that the longitudinal motion and intramural shear are important overlooked mechanisms in the cardiovascular system. The causes and control of the longitudinal motion av the arterial wall is however still large unknown. In a recent review potential factors involved are summarized and discussed (Athaide et al., 2022).

Studies on the longitudinal motion of the arterial wall in humans are so far only performed at rest, and the normal response of the longitudinal motion to physical activity in humans has not been assessed. Thus, the magnitude and nature of putative changes in longitudinal motion of the arterial wall during physical activity in humans remain unknown. To gain increased knowledge of arterial function and vascular‐ventricular interaction it is important to extend studies to situations where the body is physically active.

The aim of this study was to explore putative changes in longitudinal motion of the common carotid artery in healthy subjects in response to submaximal physical activity. For this purpose, the longitudinal motion and the diameter change of the right CCA were noninvasively recorded at rest and during supine exercise on bicycle using ultrasound and measured by an in‐house developed method. To study putative differences with aging (due to the fact that other measures of large artery function, such as the stiffness of the common carotid artery, are age‐dependent), the experiments were performed on two different age‐groups, one younger and one older. Arterial wall motion measurements during physical activity are a challenging task. To remove low frequency movements of the upper part of the body caused by breathing an averaging algorithm was also developed and evaluated.

## 2 MATERIAL AND METHODS

### 2.1 Material

The experiments were performed on 40 healthy subjects (10 young men, age range 22–35 years, mean age 27 ± 4 years; 10 young women, age range 22–34 years, mean age 28 ± 4 years; 10 older men, age range 58–67 years, mean age 62 ± 3 years; and 10 older women, age range 55–68 years, mean age 63 ± 4 years). None of the subjects reported previous cardiopulmonary disease, diabetes or smoking, and all were free from current medication (inclusion criteria). Blood pressure at rest >140/90 at the examination was an exclusion criterion (the study was performed in 2015 using the definition of hypertension at that time). The Ethics Committee, Lund University, approved the study protocol and written informed consent was obtained from all subjects before the study.

### 2.2 Study protocol ‐ exercise

The influence of exercise was studied using a supine bicycle (e‐Bike, General Electrics) at an initial workload of 30 W with 20 W increases in resistance at 2‐min intervals. ECG was monitored continuously. In addition, auscultatory blood pressure was measured in the left upper arm at rest and at each level of exercise (see below), as well as every minute during at least 5 min after exercise was finished. The Borg Scale (Borg, 1982) for ratings of perceived exertion (RPE) was used to rate the intensity of the physical activity. The participant was told to stop biking when the perceived exertion was rated as somewhat hard—hard (heavy); RPE 13–15/20. However, in some cases the participant was told to stop earlier, since we during ultrasound scanning realized that the recordings showed obvious out‐of‐ plane movements, due to muscular activity and breathing, and were considered to not allow measurements of the longitudinal motion.

The subjects were positioned on the supine bicycle, the equipment for blood pressure and heart frequency measurements were applied, and the subject was familiarized with supine cycling. The subject then had to rest for at least 10 min on the bike (with their legs resting on the bike/pedals). The room was kept quiet. Room temperature was kept at 21–23°C.

After at least 10 min of rest (see above) ultrasound registrations of the right common carotid artery were performed (baseline). Four consecutive registrations of about 5 s were recorded (two while normally breathing and two when the subject had gently stopped breathing for a few seconds). Immediately after, the peak systolic velocity in the CCA was recorded using pulse wave Doppler, and at the same time auscultatory blood pressure was measured. To ensure reproducibility of images at all stages of exercise, the position of the ultrasound transducer during baseline assessment was marked on the participant's neck. This enabled rapid identification of the same site of the common carotid artery to be imaged during and after exercise.

After the initial rest, the subject started biking at an initial workload of 30 W. During the end of the last minute at each level of workload the participant were asked to stop biking for about 10 s (with their feet still on the pedals) and ultrasound recordings of the carotid artery were performed (one‐two registrations of about 5 s), immediately followed by Doppler recordings of the blood flow velocity at the same site. At the same time as the ultrasound recordings were performed auscultatory blood pressure was measured. Then the participant was told to continue biking, and the workload was step wise increased 20 W until the desired degree of exertion was reached (see above). Breathing was not halted during the measurements. The participant then had to rest on the bike and ultrasound and Doppler registrations of the carotid artery, as well as blood pressure measurements, were again performed every minute during at least 5 min.

The exercise stimulus, expressed as % age‐predicted heart rate maximum (HR_%_), was calculated based on the age‐predicted heart rate maximum (Tanaka et al., 2001) using

(1) $${\mathrm{HR}}_{\%}=\left(\frac{\mathrm{HR}}{208-\left(0.7*y\right)}\right)\times 100$$

where *y* is the age and *HR*, the heart rate at peak exercise of the subject.

### 2.3 Ultrasonic measurements of the arterial wall movements

**FIGURE 1:**
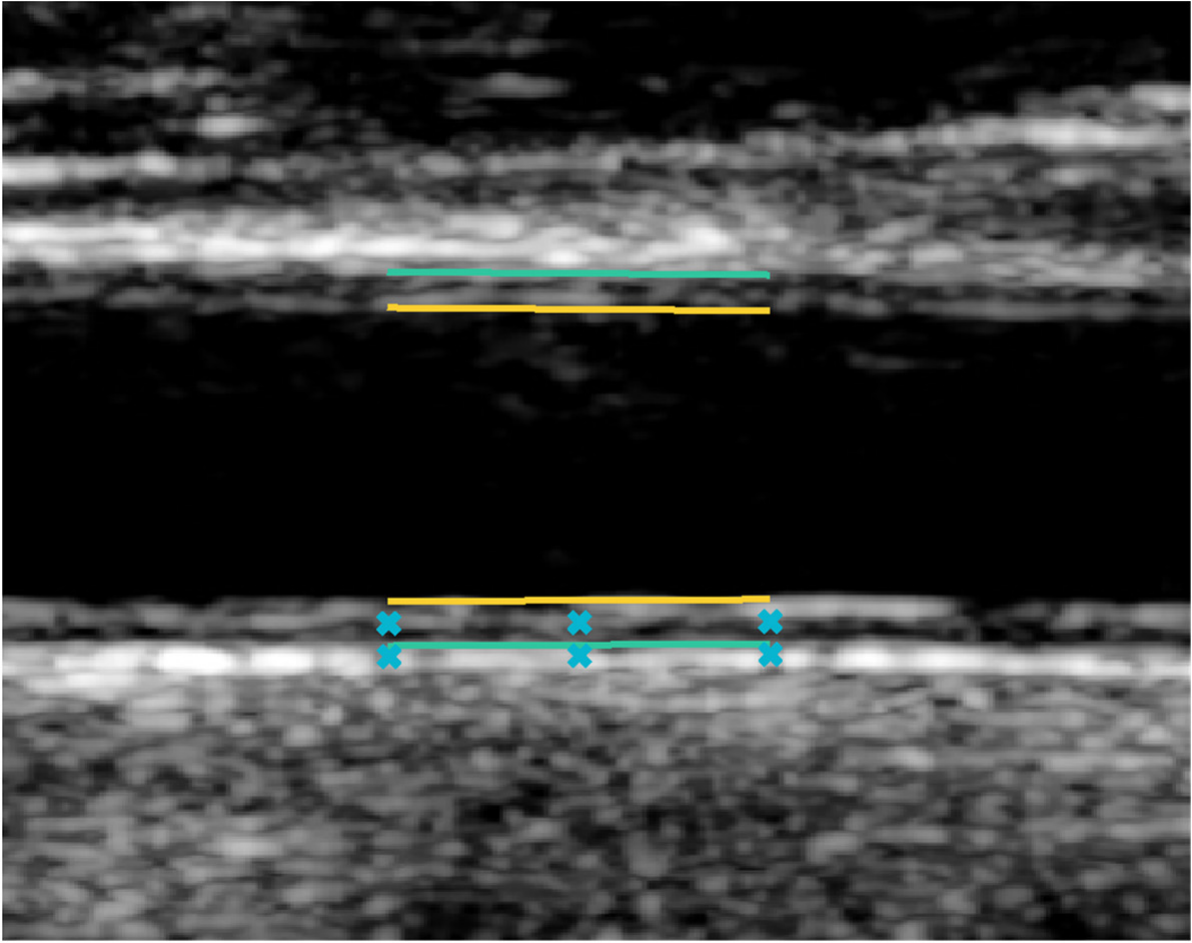
Ultrasound image of the common carotid artery. The yellow lines mark the demarcations between lumen and tunica intima and the green lines the demarcation between media and adventitia. The lines are 5 mm wide. In this study we measure the longitudinal movement at the far wall between the yellow and the green lines, and the shear strain between the blue crosses at intima‐media and adventitial layers.

The ultrasound examinations were performed using a Philips Epic 7 (Philips Medical System, Bothell, WA, USA), equipped with a 38 mm 3–12 MHz linear array transducer (L12‐3). The measurements were performed on the right common carotid artery approximately 2 cm proximal to the carotid bifurcation. The artery was scanned in the longitudinal direction, oriented horizontally in the image (Figure 1). One transmit focus was used and the persistence function turned off to avoid averaging between frames. No image processing was applied. As described above one or two consecutive registrations of about 5 s were recorded. The same experienced ultrasound technician performed all ultrasound registrations.

The image data were transferred to a PC for post‐processing. Longitudinal motion of the intima‐media complex, and, when possible (see below) of the adventitial region, as well as the minimum lumen diameter (at end‐diastole), distension and intima‐media thickness (IMT) were automatically measured using an in‐house developed image analysis software based on Nilsson et al., 2013 and Nilsson et al., 2014 using MATLAB (The MathWorks Inc.). To measure intramural shear strain of the arterial wall, the longitudinal motion was measured in both the intima‐media and the adventitial regions, respectively, and the intramural shear strain angle (γ) was calculated using

(2) γ=arctanϕ*180π

where

(3) $$\phi =\frac{\left[{\text{LMov}}_{\mathrm{IM}}-{\text{LMov}}_{\mathrm{IM}\left(\mathrm{ed}\right)}\right]-\left[{\text{LMov}}_{\mathrm{Adv}}-{\text{LMov}}_{\mathrm{Adv}\left(\mathrm{ed}\right)}\right]}{d}$$

and *LMov* is longitudinal movement, *IM* is intima‐media, *Adv* is adventitia, *ed* is end diastole, and *d* the radial distance between the two measurement positions (see also Figure 1). The maximum shearing angle during the first antegrade movement during systole (phase A) is presented (see below).

**FIGURE 2:**
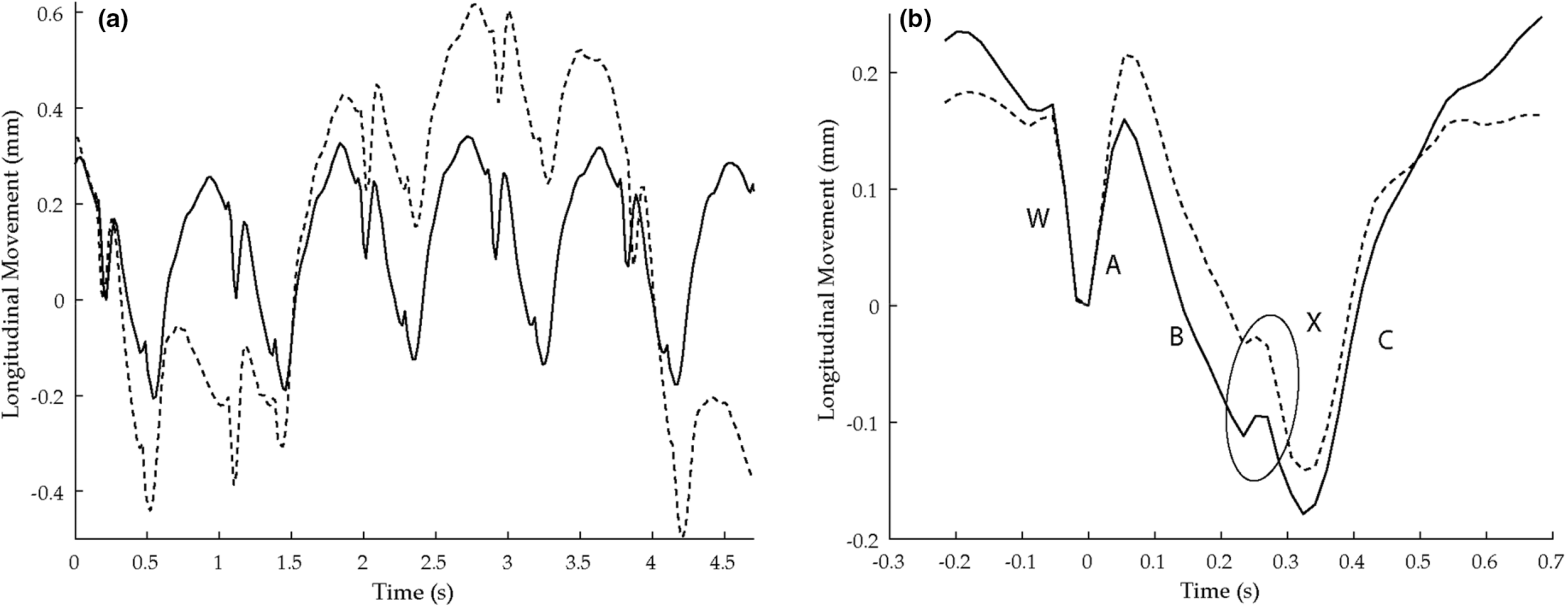
(a) The longitudinal movement of the intima‐media complex during normal breathing (dashed) and when the subject (64‐year‐old women) has gentle stopped breathing during five cardiac cycles at rest (solid line). Note that breathing causes a low frequency movement that is superimposed on the distinct multi‐phasic movement. (b) The corresponding averaged movement curves. Note that the novel phase averaging algorithm can remove the superimposed movement. End‐diastole is denoted by the origin of coordinates, based on the simultaneously measured diameter change curve. Five different phases of displacement are identified and marked by A, B, C, X and W, respectively (for description see the Result section and Cinthio et al., 2018). In short, A denotes the first antegrade displacement (i.e., in the direction of blood flow) when the diameter starts to increase, followed by a retrograde displacement (B), that is, in the direction opposite to blood flow.

Breathing introduces a global low frequency movement of the upper part of the body that overlayed the longitudinal motion of the arterial wall (Figure 2a). To enable measurements of the longitudinal motion of the arterial wall during breathing, the different cardiac cycles were averaged (Figure 2b) after first being individually phase shifted to match each other (note that *phase* in the context relates to the signals time domain phase and differs from its denotation in other parts of this paper): First, the longitudinal motion curve was differentiated, and Hilbert transformed into a complex signal. The timing of end‐diastole in each cardiac cycle was identified using the diameter curve. These locations were then used to divide the complex longitudinal movement curve into equally long cardiac cycles. Then, the average phase shift (APS) between each cardiac cycle and all others was calculated using Equation 4:

(4) $$\mathrm{APS}\left(n\right)=\frac{1}{N}\sum_{k=1}^{N}\left(\tan^{-1}\;\left(\sum_{i=1}^{M}C_{n}\left(i\right)*\hat{C_{k}}\left(i\right)\;\right)\right)$$

where, *C*_*k*_ is the k:th out of *N* cardiac cycles, *M* is the number of samples used in a cardiac cycle and *“^”* denotes the conjugate. To improve stability, only the systolic part of the cardiac cycle was used for the APS. Each (full) cardiac cycle was then phase shifted with its corresponding APS. All cardiac cycles were averaged before calculating the real part. Finally, the resulting longitudinal motion was obtained by taking the cumulative sum of this average. Also, to remove drift, since the acquisition did not start and end at the same phase of the breathing cycle, a fitted straight line was subtracted from the result. This averaging procedure also suppresses erroneous motion that do not occur at the same time in every cardiac cycle.

In the averaged longitudinal movement curve different phases of longitudinal displacement were identified and measured as recently described (Cinthio et al., 2018), Figure 2b, see also below. End‐diastole at the measurement location was identified using the diameter change curve. Thereafter, the start of the first antegrade phase of the longitudinal movement in early systole (phase A), or if absent, the start of phase W, was identified followed by the other phases of movement.

In addition, the diameter change, the relative diameter change (the fractional diameter change; arterial strain) was measured/calculated. Arterial strain (AS) was defined as:

(5) $$\mathrm{AS}=\frac{d_{\mathrm{sys}}-d_{\mathrm{dia}}}{d_{\mathrm{dia}}}$$

where *d* is lumen diameter, *sys* is systole, and *dia* is diastole.

Further, the distensibility of the arterial was estimated and expressed as the distensibility coefficient DC defined as:

(6) $$\mathrm{DC}=\frac{\mathrm{AS}}{P_{\mathrm{sys}}-P_{\mathrm{dia}}}$$

where *P* is auscultatory blood pressure.

Also, the CCA peak systolic velocity (v_sys_) was measured at rest and at each workload (see above) using pulse wave Doppler (the peak Doppler envelope). The wall shear stress (WSS) was estimated using v_sys_ and d_sys_ using Equation 7 (Papaioannou & Stefandis, 2005):

(7) $$\mathrm{WSS}=\frac{8\eta v_{\mathrm{sys}}}{d_{\mathrm{sys}}}$$

where η is the viscosity and defined as 0.0035 Ns/m^2^.

### 2.4 Evaluation of the new averaging algorithm

To evaluate the averaging algorithm, the longitudinal movement of the arterial wall of the right common carotid artery were analyzed in 20 of the subjects (10 men and 10 women, age range 22–68 years) at rest, both during normal breathing and when the subjects have gently stopped breathing for a few seconds. The agreement was evaluated by estimating intraclass correlation coefficient (Model: two‐way mixed effects. Type: Multiple measurements, Definition: absolute agreement) (Koo et al., 2016) and Bland–Altman (Bland Altman 1986) using MATLAB (The MathWorks Inc).

### 2.5 Statistics

Data are presented as the mean value ± SD, unless otherwise stated. Independent samples *T*‐test for Equality of Means, Independent‐Samples Mann–Whitney *U*‐test, Paired samples *T*‐test, and Related‐Samples Wilcoxon Signed Rank Test, respectively, were used for comparisons. The Shapiro–Wilk test was used for assessing normality. These statistical analyses were performed using SPSS (IBM SPSS Statistics for Windows, Version 28). Relationships between variables were assessed by Spearman rank order correlation coefficient and, in some cases, Pearson's product moment correlation, using MATLAB (The MathWorks Inc.). *p* < 0.05 was taken as significant.

## 3 RESULTS

### 3.1 Baseline

Clinical characteristics and measurements at baseline, that is, at rest before the bicycle was started are presented in Table 1. As expected, systolic and diastolic blood pressures were significantly higher (*p* ≤ 0.001 and *p* = 0.004) in the older age group than in the young. Further, CCA strain (the relative diameter change) was lower in the older age group than in the young (*p* = 0.001). Further, as expected, the distensibility coefficient (DC) was lower and IMT was larger in the older age‐group than in the young (*p* ≤ 0.001 and *p* ≤ 0.0001, respectively). In addition, CCA peak systolic velocity was significantly higher in the young than in the older age‐group (*p* ≤ 0.001). The CCA end‐diastolic diameter did not significantly differ between the young and older age‐group (i.e., when men and women were analyzed together).

**TABLE 1:** Clinical characteristics and baseline measurements

	Young subjects	Older subjects	
Number of subjects	20	19	
Age (years)	27.9 ± 4.0	62.6 ± 3.7	***
Height (m)	1.73 ± 0.06	1.74 ± 0.10	
Weight (kg)	68.0 ± 8.8	74.6 ± 15.0	
BMI	22.8 ± 2.2	24.4 ± 2.6	*
Systolic pressure (mm Hg)	113 ± 13	129 ± 10	***
Diastolic pressure (mm Hg)	73 ± 10	82 ± 8	**
Pulse pressure (mm Hg)	40 ± 13	47 ± 9	
Heart rate (beats/minute)	68 ± 12	64 ± 10	
IMT (μm)	554 ± 64	780 ± 182	***
Minimal lumen diameter (μm)	5623 ± 521	5868 ± 519	
Lumen distension (μm)	733 ± 132	470 ± 123	***
Arterial strain (%)	13.1 ± 2.5	8.0 ± 2.0	***
DC (10 e‐5 1/Pa)	5.6 ± 2.4	2.7 ± 0.8	***
Maximal systolic blood flow velocity (cm/s)	93 ± 15	75 ± 14	***
Maximal shear stress (Pa)	4.1 ± 0.9	3.3 ± 0.8	**

*Note*: All data, except frequencies, are presented as mean ± SD. *indicates significant difference between young and older subjects; *<0.05, **<0.01, ***<0.001.

#### 3.1.1 Longitudinal displacement

**FIGURE 3:**
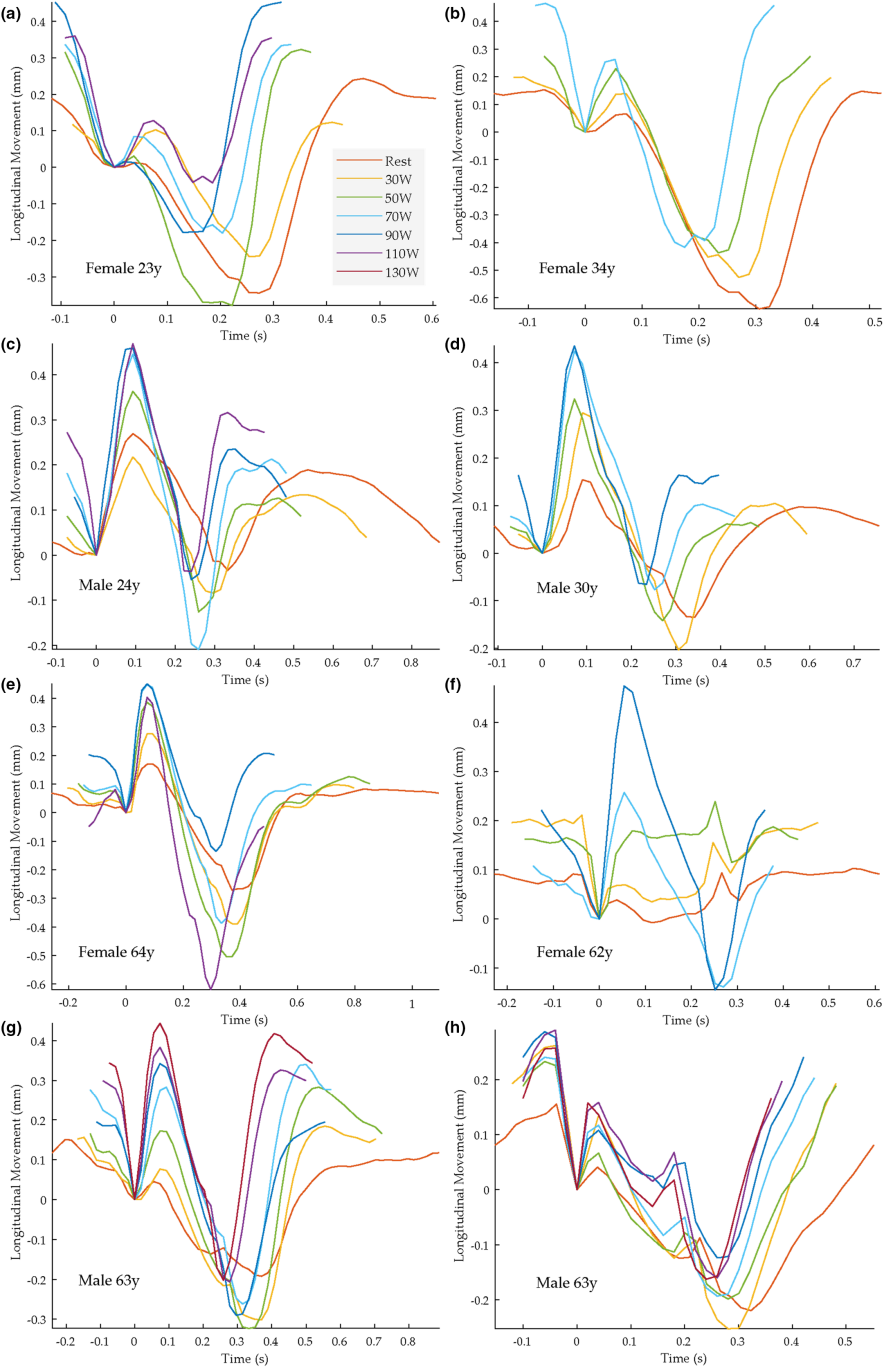
The longitudinal movement of the intima‐media complex during rest and up to six different loads (30, 50, 70, 90, 110 and 130 W) in eight different subjects. The different colors denote different loads (se explanations in panel a); thus, the red curve show the motion at rest. End‐diastole at the measurement site is denoted by origin of coordinates (based on the simultaneously measured diameter change curve). Note the dramatically increase of longitudinal movement of the arterial wall during exercise.

At baseline, that is, at rest before the bicycle was started, a distinct complex bidirectional multiphasic longitudinal motion of the intima‐media complex of the carotid artery was seen in all subjects during the cardiac cycle. As previously reported (Ahlgren, Cinthio, Persson et al., 2012; Cinthio et al., 2018) the pattern of longitudinal motion during the cardiac cycle showed different patterns. In the youngest age‐group, in both men and women, the most common pattern was a pattern showing a first antegrade displacement (i.e., in the direction of blood flow) when the diameter started to increase (in this study denoted A), followed by a retrograde displacement (B), that is, in the direction opposite to blood flow, which in turn was followed by a second antegrade displacement (C), before the wall returned to its initial position. Typical examples of tracings of the longitudinal motion in young subjects are seen in Figure 3a–d, red curves (i.e., at rest). The ratio between the first (A) and second (B) distinct displacement varied between individuals, thus, in some subjects there was no or a very small A, and the pattern of motion was predominantly backward oriented (Figures 3a,b, red curve).

**FIGURE 4:**
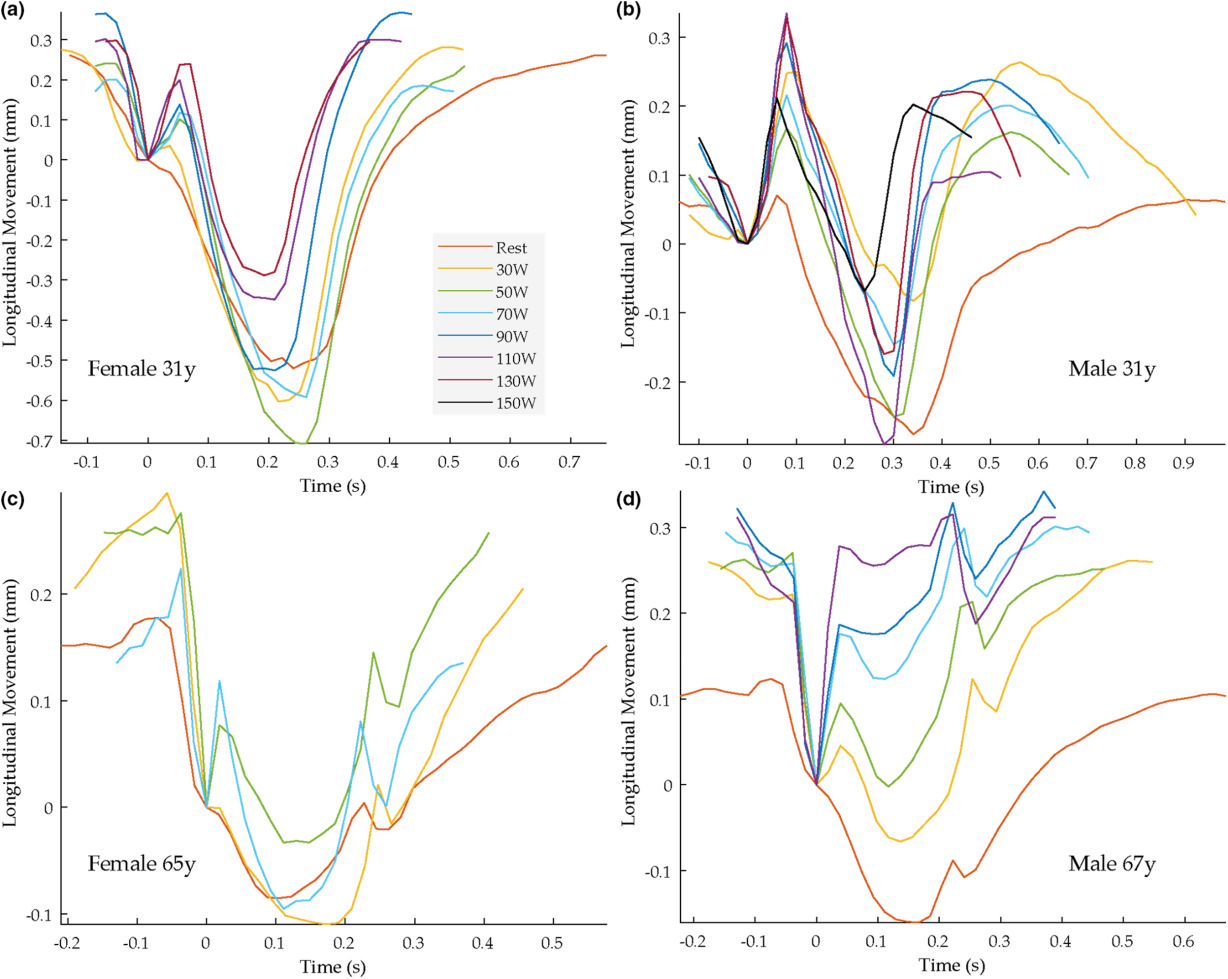
The longitudinal movement of the intima‐media complex during rest and up to seven different loads (30, 50, 70, 90, 110, 130 and 150 W) in four different subjects. The different colors denote different loads (see explanations in panel a), thus the red curve show the motion at rest. End‐diastole at the measurement site is denoted by origin of coordinates (based on the simultaneously measured diameter change curve). Note the absent of a phase A at rest in three of the subjects (panel a, c, d). During exercise the phase A show up and increases dramatically.

In the older age groups, other patterns of longitudinal motion were also present. As recently described (Cinthio et al., 2018) one prominent finding with aging is that a distinct antegrade displacement (in this study denoted X, Figure 2b) become clearly visible at the time, or close to, when the dicrotic notch is seen in the diameter change curve. Two examples of common patterns seen in the older age‐group are shown in Figure 3f,h (red curves), in these note the prominent phase X. Further examples of the basic pattern of motion seen at rest are given in Figure 4 (red curves). Note that in the subjects in Figure 4a,c,d (red curves) no distinct antegrade displacement is seen in early systole.

The amplitude of the different phases of longitudinal motion, as well as the maximum longitudinal displacement (LMovMax; peak‐to‐peak value), that is, the difference between the most antegrade and the most retrograde position of the intima‐media complex during the cardiac cycle, irrespective of phase, at baseline (rest) for both young and older subjects are given in Table 2. When the values of the young were compared to those of the older ones no significant difference in the amplitude of LMovMax; A or B, respectively, was seen. The amplitudes of W and X were significantly larger (*p* = 0.028 and *p* < 0.001, respectively) in the older age group, whereas C was significantly smaller (*p* < 0.001).

**TABLE 2:** Longitudinal displacement at rest

	Young subjects	Older subjects	
Max (μm)	473 ± 233	349 ± 130	
A (μm)	133 ± 133	99 ± 116	
B (μm)	374 ± 200	252 ± 154	
C (μm)	424 ± 250	182 ± 130	***
W (μm)	65 ± 77	124 ± 96	*
X (μm)	2 ± 5	45 ± 36	***

*Note*: All data are presented as mean ± SD. *indicates significant difference between young and older subjects; *<0.05, **<0.01, ***<0.001.

### 3.2 Exercise

During exercise heart rate and blood pressure increased as expected (*p* < 0.001 and *p* < 0.001, respectively). Further, CCA blood flow systolic velocity as well as CCA shear stress and distension increased significantly (*p* < 0.001, *p* < 0.001 and *p* < 0.001, respectively). Further, CCA strain (the relative diameter change) increased (*p* < 0.001), and DC decreased (*p* < 0.001). No significant change in CCA end‐diastolic diameter or IMT was seen. The measurements at peak exercise for both young and older subjects are summarized in Table 3. The exercise stimulus expressed as % age‐predicted heart rate maximum did not differ between young and older subjects.

**TABLE 3:** Measurements at peak exercise

	Young subjects	Older subjects	
Systolic pressure (mm Hg)	154 ± 14^†††^	177 ± 18^†††^	***
Diastolic pressure (mm Hg)	64 ± 10^†^	77 ± 12^†^	**
Pulse pressure (mm Hg)	90 ± 16^†††^	100 ± 15^†††^	*
Heart rate (beats/minute)	119 ± 17^†††^	111 ± 14^†††^	
IMT (μm)	541 ± 52	768 ± 100	***
Minimal lumen diameter (μm)	5544 ± 519	5868 ± 519^†^	**
Lumen distension (μm)	1064 ± 235^†††^	663 ± 225^†††^	***
Arterial strain (%)	19.3 ± 4.3^†††^	10.9 ± 3.6^†††^	***
DC (10 e‐5 1/Pa)	3.3 ± 0.8^†††^	1.7 ± 0.6^†††^	***
Maximal systolic blood flow velocity (cm/s)	133 ± 25^†††^	94 ± 17^†††^	***
Maximal shear stress (Pa)	5.7 ± 1.4^†††^	3.0 ± 1.0^†††^	***
Peak workload (W)	114 ± 19^†††^	112 ± 19^†††^	
Age‐predicted heart rate maximum (%)	63 ± 9^†††^	67 ± 10^†††^	

*Note*: All data are presented as mean ± SD. †indicates significant difference between rest and exercise; ^†^<0.05, ^††^<0.01, ^†††^<0.001. *indicates significant difference between young and older subjects at peak exercise; *<0.05, **<0.01, ***<0.001.

#### 3.2.1 Longitudinal motion

The longitudinal motion of the intima‐media complex of the carotid artery underwent marked changes in response to physical activity, already at low load, with most evident a marked increase of the first antegrade displacement in early systole (A) (*p* < 0.001). In the subjects (*n* = 33) that had a distinct A at rest (baseline) the magnitude of this phase increased by mean 2565% (range −100%–68602%), although in five young subjects A seemed to decrease, or stay at approximately the same amplitude, during exercise. For comparison the distension increased by mean 44% (range 5%–91%). Typical examples of the changes in longitudinal motion during exercise in subjects having a distinct A at rest are seen in Figure 3a–h and in Figure 4b. In the subjects that did not have a distinct A at rest (*n* = 6), a distinct phase A became clearly apparent and increased markedly during exercise in five of them (mean 106 μm, range 14–237); for examples see Figure 4a,c,d. However, interestingly, in one of these subjects (a 64‐year‐old man) no evident appearance of A during exercise was seen.

When young and older subjects were analyzed separately there was a significant increase of A in both young and older subjects (young *p* = 0.04, older *p* < 0.001). As mentioned above, in five of the young subjects A seemed to decrease or stay at approximately the same amplitude during the physical activity performed. Regarding three of these subjects, interestingly, they had by far the largest A at baseline.

Also, B, the retrograde motion following A, increased significantly during exercise (*p* < 0.001). This was also seen when young and older subjects were analyzed separately (*p* = 0.003 and *p* = 0.022, respectively).

The antegrade phase C showed no clear trends (and C was often somewhat hard to measure due to its indistinct termination).

W, the distinct retrograde motion before the onset of A, increased significantly (*p* < 0.001). This was also seen when young and older subjects were analyzed separately (*p* = 0.011 and *p* < 0.001, respectively).

The displacement of phase X seemed to be much affected by the behavior of B; X seemed to decrease when B increases, and therefore analyses of the individual change in X are difficult. When analyzed as one group the amplitude of X did not change significantly.

LMovMax increased significantly during exercise *p* < 0.001. This was also seen when young and older subjects were analyzed separately (*p* < 0.001 and *p* = 0.011, respectively).

The amplitudes of the longitudinal motion at peak exercise are summarized in Table 4. The group percentage changes of the measured parameters as compared to baseline are summarized in Table 5. Note that the group percentage increase of phase A was two to three times larger than for example systolic pressure, lumen distension, and arterial strain. Also note that the group percentage increase of A was larger in the older age group than in the young (young mean 61% vs. older mean 151%).

**TABLE 4:** Longitudinal displacement at peak exercise

	Young subjects	Older subjects	
Max (μm)	635 ± 210^†††^	537 ± 240^†^	
A (μm)	214 ± 159^†^	251 ± 154^†††^	
B (μm)	517 ± 225^††^	429 ± 296^†^	
C (μm)	403 ± 231	218 ± 189	**
W (μm)	143 ± 111^†^	231 ± 56^†††^	**
X (μm)	2 ± 5	48 ± 74	**

*Note*: All data are presented as mean ± SD. ^†^indicates significant difference between rest and exercise; ^†^<0.05, ^††^<0.01,^†††^<0.001. *indicates significant difference between young and older subjects at peak exercise; *<0.05, **<0.01, ***<0.001.

**TABLE 5:** Group percentage change in measured parameters between rest and peak exercise for all subjects, as well as when the groups of young and older subjects were analyzed separately

	All subjects	Young subjects	Older subjects
Max (%)	43^†††^	34^†††^	54^†††^
A (%)	100^†††^	61^†^	151^†††^
B (%)	51^†††^	38^††^	70^†^
C (%)	2	−5	19
W (%)	98^†††^	120^†^	87^†††^
X (%)	7	−16	8
Systolic pressure (%)	37^†††^	36^†††^	37^†††^
Minimal lumen diameter (%)	2	−1	5^†^
Lumen distension (%)	43^†††^	45^†††^	40^†††^
Arterial strain (%)	43^†††^	48^†††^	35^†††^
DC (%)	−39^†††^	−41^†††^	−36^†††^
Maximal systolic blood flow velocity (%)	35^†††^	42^†††^	26^†††^
Maximal shear stress (%)	31^†††^	40^†††^	20^†††^

*Note*: † indicates significant percentage change between rest and peak exercise; ^†^<0.05, ^††^<0.01, ^†††^<0.001.

Due to the changes in amplitudes and especially the marked changes of A, the basic pattern of motion, that is, the relations between the different phases, changed in all subjects.

#### 3.2.2 Longitudinal motion and relations to blood pressure

**FIGURE 5:**
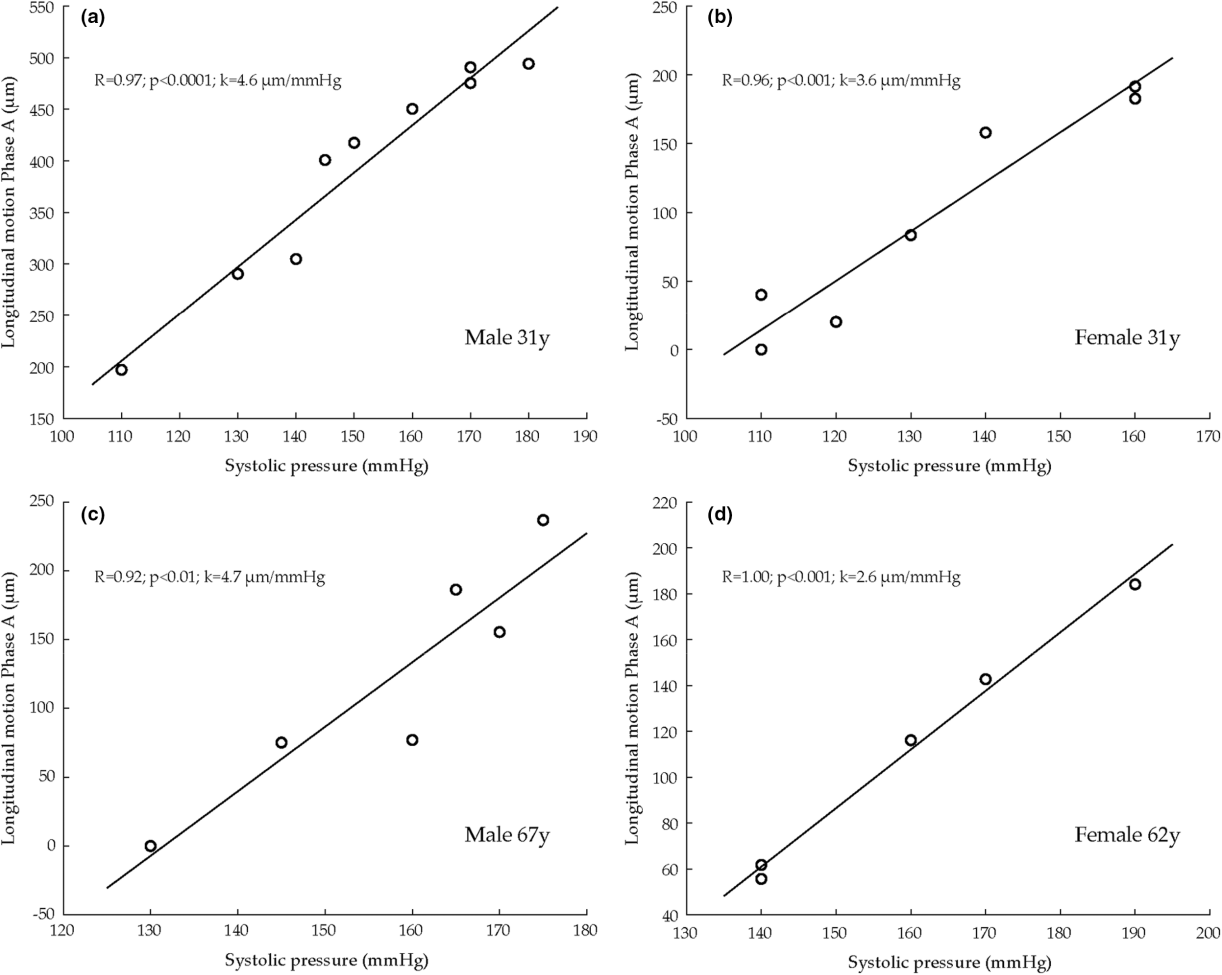
Examples of individuals in whom the correlation between the amplitude of A and systolic blood pressure during exercise was remarkably strong. Note the *r*‐values close to one.

There was a significant positive correlation between the amplitude of A and systolic blood pressure (*r* = 0.47, *p* < 0.00001). In several individuals the correlation between systolic blood pressure and the amplitude of A was remarkable strong; for examples see Figure 5a–d. Note the *r*‐values close to one in some individuals (10 of 40 individuals showed significant correlations with *r*‐values between 0.8–1.0). However, as stated above, in a few individuals A did not increase during the experiment. When young and older subjects were analyzed separately the significant correlation between the amplitude of A and systolic blood pressure remained (young *r* = 0.41, *p* < 0.00001); (older *r* = 0.51, *p* < 0.00001).

There was a significant, but weaker, positive correlation between the amplitude of B and systolic pressure (*r* = 0.21, *p* = 0.001). No significant correlation between the amplitude of C and systolic pressure was seen. There was a significant positive correlation between the amplitude of W and systolic blood pressure (*r* = 0.39, *p* < 0.00001). This correlation remained also when young and older subjects were analyzed separately (young *r* = 0.34, *p* < 0.00001; older *r* = 0.41, *p* < 0.00001). There was a weak negative correlation between the amplitude of X and systolic blood pressure (*r* = −0.16, *p* = 0.02).

There was a significant correlation between systolic pressure and LMovMax (*r* = 0.22, *p* < 0.001). When young and older subjects were analyzed separately this correlation remained in young (young *r* = 0.31, *p* = 0.0006) but not in older subjects.

#### 3.2.3 Longitudinal motion and relation to CCA blood flow velocity and wall shear stress

No significant correlation between the maximal systolic blood flow velocity and the amplitude or velocity of A, respectively, was seen. Further, no significant correlation between maximal wall shear stress and the amplitude or velocity of A, respectively, was seen.

#### 3.2.4 Longitudinal displacement and relations to distension

There were significant correlations between CCA distension (diameter change) and all phases of longitudinal movement except W (A *r* = 0.24, *p* = 0.0003; B *r* = 0.25, *p* = 0.0001, C *r* = 0.18, *p* = 0.006; X *r* = −0.28, *p* < 0.0001, LMovMax *r* = 0.25, *p* = 0.0002).

#### 3.2.5 Intramural shear strain

In a subgroup of the investigated subjects (*n* = 18; 9 young, 9 older) intramural shear strain was estimated. Thus, the longitudinal motion of not only the intima‐media complex but also the longitudinal motion of the adventitial region was analyzed, focusing on phase A, that is, the difference in longitudinal motion between A of the intima media complex and A of the adventitial region. To measure this during exercise is an especially challenging task (see Discussion and Methods) and only the subjects with the very best quality recordings were analyzed. Further, subjects with a distinct A were chosen. Intramural shear strain increased significantly during exercise (when analyzed at 90 W *p* = 0.01; when analyzed at peak exercise *p* = 0.004). The percentage mean increase of intramural shear strain were 177% at 90 W and 296% at peak exercise.

When young and older subjects were analyzed separately the mean increase was significantly larger in the older group than in the young at 90 W (*p* = 0.03), but not at peak exercise (*p* = 0.09) (Table 6). The group percentage increase was 31% at 90 W and 143% at peak exercise in the young. In the older age group, the group percentage increase was 549% at 90 W and 682% at peak exercise. The reason for us to present shear strain not only at peak exercise, but also at 90 W, was the fact that it in some subjects it was not possible to measure shear strain at their highest workloads, and there were fewer adequate measurements at higher loads. It was however possible to measure the intramural shear strain up to 90 W in all chosen individuals. Interestingly, in one young man it was possible to adequately measure shear strain up to 170 W and in this young subject the intramural shear strain increased dramatically at 130 W: from 2–3° at 70–110 W, to 12–27° at 130–170 W.

**TABLE 6:** Intramural shear strain at Phase A at rest, at 90 W, and at peak exercise in the subgroup of subjects analyzed

	Young subjects	Older subjects	
Number of subjects	9	9	
Shear strain at rest (°)	2.8 ± 4.3	1.1 ± 1.0	
Shear strain at 90 W (°)	3.6 ± 4.2	7.1 ± 4.6^††^	*
Shear strain at peak exercise (°)	6.8 ± 8.2	8.6 ± 5.6^††^	
Peak workload (W)	119 ± 27^†††^	112 ± 16^†††^	
Age‐predicted heart rate maximum (%)	68 ± 8^†††^	67 ± 7^†††^	

*Note*: All data except frequencies are presented as mean ± SD. †indicates significant difference between rest and exercise; ^†^<0.05, ^††^<0.01, ^†††^<0.001. *indicates significant difference between young and older subjects; *<0.05, **<0.01, ***<0.001.

**FIGURE 6:**
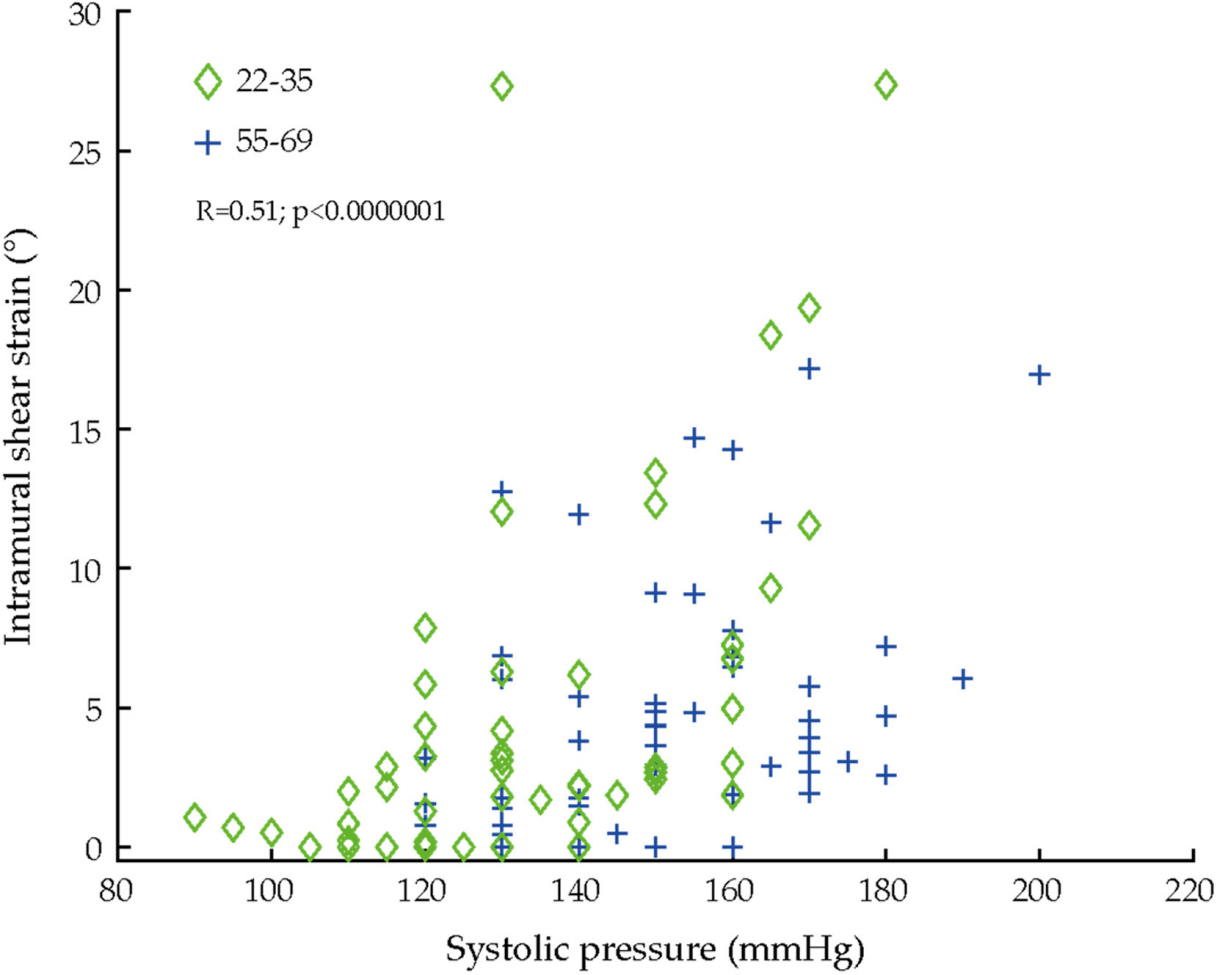
Intramural shear strain in relation to systolic pressure in the analyzed subgroup (*n* = 18). It is to be noted that in some individuals (*n* = 5) we could not adequately measure shear strain at their highest workloads, despite this a significant correlation to blood pressure is seen.

Figure 6 show intramural shear strain between the intima‐media complex and the adventitial region for A in relation to systolic blood pressure in the analyzed individuals. There was a significant correlation between systolic blood pressure and shear strain (*r* = 0.51, *p* < 0.0000001).

#### 3.2.6 Evaluation of the new ultrasonic method

The averaging and compensation algorithm was able to compensate for the low frequency motion due to breathing (see Figure 2). The intraclass correlation coefficient for the algorithm showed excellent agreement between measurement obtained during normal breathing, and when the subjects had gently stopped breathing, and ranged between 0.90–0.96 (*p* < 10–5). The mean differences for LMovMax and the different phases of movement were 2 ± 94, 11 ± 61, −3 ± 64, 2 ± 81, 7 ± 43, and 2 ± 17 μm, respectively.

## 4 DISCUSSION

This is the first study to examine the response of the longitudinal motion of the arterial wall to physical activity in humans. We show that the complex multiphasic, bidirectional longitudinal motion of the intima‐media of the common carotid artery (CCA) undergoes marked changes in response to exercise already at low workload. The most prominent change is an increase (or emergence) of the antegrade displacement (A), that is, in the direction of blood flow, seen in early systole, that is, when the artery begins to distend. As expected, due to the low workload, the increase in heart rate and blood pressure in this study are moderate. Despite this, significant correlations between longitudinal displacement and blood pressure were seen, especially between the first antegrade motion A and systolic blood pressure, and in some subjects the correlation was remarkably strong. In contrast, no correlation between CCA max systolic blood flow velocity or shear stress, respectively, and the antegrade motion A was seen. We hypothesize that the increase in longitudinal displacement of the arterial wall, especially the increase in the first antegrade motion (A), is an important overlooked “cushioning” mechanism in response to increase in blood pressure and stroke volume to reduce forces/stresses within/at the wall as discussed below. Further, it seems reasonable that the changes in longitudinal motion can have impact on the circulation of the vasa vasorum, as well as on endothelial function and the smooth muscle cells and the extracellular matrix components of the media as discussed below.

To study the longitudinal motion of the carotid artery wall during physical activity is a challenging task given that the vessel wall is only a few mm thin, the movements are less than a mm, and the subjects investigated are moving. To minimize these problems, we have chosen to study the response to exercise using a supine bike with a soft holder to stabilize the head, and the upper part of the body resting on the bike, thereby minimizing disturbing movements of the upper part of the body during the ultrasound registrations. In this study, we have also chosen to increase workload step wise with 20 W every second minute. One reason for this was to ensure enough time to achieve several very good quality ultrasonic recordings of the longitudinal motion at each step of workload. To be able to measure the longitudinal motion of the arterial wall, it is critical to achieve very high‐quality ultrasonic recordings of the vessel wall with distinct visualization of the intima media complex and the adventitial region during the whole cardiac cycle. The Borg Scale of perceived exertion was used to rate the intensity of the physical activity (Borg, 1982), and as stated above, the participants were told to stop biking when the perceived exertion was rated as somewhat hard—hard (heavy), RPE 13–15/20. The reason for this was that, as expected, it was not possible to measure the longitudinal motion when the subject was heavy breathing and working hard, due to disturbing movements of the body. To further improve our possibilities to study the longitudinal vessel wall motion during physical activity, we have developed an averaging algorithm to remove low frequency movements of the body, for example, induced by breathing. This includes both phase shifting of multiple cardiac cycles before averaging and drift compensation. The evaluation of the algorithm showed a convincing ability to extract the longitudinal motion from curves that contains low frequency breathing motion artifacts.

Physical activity is associated with increased levels of circulating catecholamines. In healthy subjects the most obvious response to exercise is increase in heart rate, stroke volume and systolic and pulse pressure. In the present study we show that apart from a marked increase, or emergence of, an antegrade motion in early systole (A) there is also an increase in the retrograde phase B, following A, and of the retrograde phase W as well as in LMovMax.

In previous experiment on the porcine carotid artery of anesthetized pigs we have shown that the longitudinal motion is profoundly influenced by adrenaline and noradrenaline, and that increase in longitudinal motion seems to be strongly related to α‐adrenoceptor activation (Ahlgren, Cinthio, Steen et al., 2012). In that study we found strong positive correlations between pulse pressure and longitudinal displacement of the intima‐media complex (Ahlgren, Cinthio, Steen et al., 2012). The porcine carotid artery is a muscular artery whereas the human carotid artery is predominantly elastic. Further, due to the different species, among other differences, the adrenoceptors may not have the same distribution, thus the response to catecholamines cannot obviously be expected to be the same. However, the findings in the present study are in line with the findings in our previous study of the porcine carotid artery although the basic pattern of motion is not the same.

This study shows that local blood flow velocity appears to be of little or no importance for the changes in longitudinal motion of the carotid artery during physical activity, and there was no significant correlation between the first antegrade longitudinal displacement and maximal wall shear stress. These findings are in line with our previous findings on studies on the porcine carotid artery (Ahlgren et al., 2015) which showed that a profound increase in longitudinal motion can take place independently of wall shear stress from the blood flow. Our present findings are also supported by a recent case study using premature ventricular contractions to study the carotid artery longitudinal wall motion (Stevens & Au, 2021). In that study forward wall motion remained unchanged despite large deviations in local blood velocity.

As previously shown the intima‐media complex, that is, the inner layers of the arterial wall, shows a larger longitudinal motion than the adventitial region, indicating presence of shear strain, and thus, shear stress within the arterial wall (Cinthio et al., 2006). In healthy subjects at rest the largest difference in movement seems to take place close to, or at, the lamina elastica externa (Nilsson et al., 2010), the demarcation between the media and the adventitia. At present there are no technique available to study if there is also a similar difference in movement between the tunica intima /intimal layer and the tunica media since it is impossible to measure the tunica intima, a single cell layer with ultrasound. Measurements of intramural shear strain are difficult and are subjected to higher variations (Cinthio & Ahlgren, 2010), since the calculation of shear strain angle is a comparison between two different measurements of the longitudinal movement, that is, ROIs are positioned at two different locations: in the intima‐media complex and the adventitial region, respectively. This is especially challenging during exercise, and in this study, we could only measure the motion of the adventitial region in a limited number of subjects during the different workloads. In our previous experiments on the carotid artery of anesthetized pigs, with simultaneous measurement of longitudinal displacement and intraarterial blood pressure, we found strong correlations between intramural shear strain and increase in blood pressure (Ahlgren, Cinthio, Steen et al., 2012). The present study shows that also in the human carotid artery increase in blood pressure is associated with increased difference in motion between the intima media complex and the adventitial region, illustrated by the increase in estimated shear strain.

Large central arteries have a function of not only being conduits, but most importantly, also to serve as a cushion (or buffer) to flow pulsations, also known as the “Windkessel function”. Simplified, the main role of arteries as cushions is to smooth out the pressure oscillations resulting from intermittent ventricular ejection. Large elastic arteries can instantaneously accommodate the volume of blood ejected from the ventricles, storing part of the stroke volume during systole, and draining this volume during diastole, thus permitting continuous perfusion of peripheral organs and tissues. The efficiency of arteries as cushions depends on the compliance and distensibility of the arterial walls and on the viscoelastic properties of the vessels. Arterial wall viscoelasticity also affects the speed of wave propagation (pulse‐wave velocity) and the timing of wave reflections. Stiffening of the arteries increases pulse‐wave velocity and may cause an earlier return of the reflected wave (Safar 1996). Increased stiffness of large central arteries has been shown to be an independent risk factor for cardiovascular mortality (Blacher et al., 1999; Laurent et al., 2001). In healthy subjects the most obvious response to exercise is increase in heart rate, stroke volume and systolic and pulse pressure. When blood pressures increase, the hemodynamic forces acting on the arterial wall increase and it seems reasonable to expect that this will increase the longitudinal motion of the wall, as seen in this study. Further, due to tethering, and because of the multilayer elastic structure of the arterial wall, it seems reasonable to expect that the inner wall layers will not exhibit the same degree of restricted movement as the outer layer, the adventitial region. Due to this, shearing forces/shear stress will be present in the wall, as seen in this study. We hypothesize that part of the hemodynamic forces is dissipated by the displacement of the various layers of the arterial wall, thereby reducing forces/stresses within/at the wall, and thus in that way having a dampening function in addition to the well‐known “Windkessel function” due to the distensibility in the circumferential direction.

An interesting question is how the increase in longitudinal motion, and the accompanying cyclic increase in intramural shear strain, during physical activity, as shown in this study, may influence the function of the arterial wall. The innermost layer of the arterial wall, the intima, consists of vascular endothelial cells. This metabolically active layer is constantly exposed to biomechanical and biochemical stimuli, and it is well established that the vascular endothelium has a crucial role in vascular biology and the development of atherosclerosis. Changes in longitudinal motion has the potential to either augment or diminish endothelial shear depending on the phase relationship to oscillatory flow (Cinthio et al., 2006; Halliwill & Minson, 2010). Furthermore, the smooth muscle cells of the media and the extracellular matrix components have proven to be capable of numerous functions considered important in the pathogenesis of vascular diseases such as atherosclerosis (Hunt et al., 2002), and the longitudinal motion and its changes during physical activity has the potential to have impact on this as well. Furthermore, the adventitia, the outermost layer of the arterial wall of larger arteries, contains small blood vessels, the vasa vasorum (“vessels of vessels”), which in the largest arteries also penetrate into the media (for reviews see Ritman & Lerman, 2007; Zhao et al., 2021), that is, passing through the area of the demonstrated changing cyclic shear strain. There is increasing evidence suggesting a role for the vasa vasorum in the development of atherosclerotic vascular disease (for review see Xu J et al. 2015), and it is reasonable to hypothesize that changes in longitudinal motion and resulting cyclic shear strain influence the circulation of the vasa vasorum possibly/probably causing transient compression of the vasa vasorum. The marked changes in longitudinal movement seen already at low workloads in this study could therefore be interesting also in the context of physical activity and its effect on cardiovascular health. The beneficial effects of physical activity on cardiovascular health are now well established (for review see Lavie et al., 2015) and it has been shown that also only low (as compared to no) physical activity have favorable effects and can improve cardiovascular health. Although many factors involved have been identified (for review see Pinckard et al., 2019), the exact mechanisms behind the beneficial effects of physical activity are unclear. Given the possible impact of the longitudinal motion on the vessel wall described above, an interesting issue is if the putative effect of increase longitudinal motion and resulting intramural shear strain during physical activity can be an overlooked factor of vital importance.

It is not known if the putative response of the longitudinal motion of the arterial wall to physical activity change during the cardiovascular aging process. In an attempt to evaluate possible influence of aging on the response to physical activity we have chosen to study two different age group, one young and one older. It is well known that in large central arteries both the diameter and the wall stiffness increase with aging, also seen in this study—at baseline the distensibility coefficient (DC) were lower in the older age group than in the young. Further, as expected blood pressures were higher in the older age‐group than in the young. Blood flow velocity in the CCA is age‐dependent (Scheel et al., 2000), and as expected maximal systolic velocity were higher in/at the young age‐group than in the older. This study cannot clearly state whether the response of the longitudinal motion to physical activity changes with aging, and to answer this question further studies are needed. However, it can be speculated that the longitudinal motion and the resulting intramural shearing are becoming more important with aging and increase in arterial stiffness. Interestingly, in this study the increase in shear strain during exercise seemed to appear at lower load in the older age group than in the young. Further, when we analyzed the ultrasound files, we noted that the five young subjects, of who had the largest longitudinal movement at rest (and also the highest distensibility [DC]), did not show as clear increase in longitudinal motion during activity as in other subjects; hypothetically because they already “have enough” buffering. Thus, findings that support our hypothesis.

It is obviously difficult to determine the forces behind the different phases of the longitudinal movement as the hemodynamic forces are so intertwined (Ahlgren, Cinthio, Steen et al., 2012 ; Athaide et al., 2022; Cinthio et al., 2006). An interesting question in this context is whether the amplitude of A is predominantly driven directly by the blood pressure or indirectly by the blood pressure via the diameter change through a longitudinal‐circumferential mechanical coupling. It was not the aim of this study to elucidate this question, but we have noticed that the correlation between that amplitude of A and the systolic blood pressure was very strong (*r* > 0.8) in 25% of the subjects and that the amplitude of A did increase several 100% in some individuals at the same time the distension did not increase more than 90% in any subjects. However, further studies are needed to elucidate this.

The participants in this study were all normotensive non‐smokers, non‐obese without any medication and were mainly recruited from our near surrounding, that is, mainly colleagues and friends, most of them probably having a healthier lifestyle than the average population. An interesting issue is if, and how, cardiovascular risk factors influence the response of the longitudinal displacement to physical activity, and this is an interesting issue for future studies.

It must be noted that the present results were obtained during supine cycling exercise and that altered hemodynamics during upright exercise may be associated with different changes in longitudinal displacements. Further, since existing methods do not allow measurements of the longitudinal displacement and intramural shear strain when the body is heavy working (due to movement of the body and large breathing artifacts) this study cannot describe the changes at high load with high heart rate and high blood pressures. In this study we have focused on the changes during physical activity and the changes post‐exercise have not been analyzed in detail. However, after 5 min of rest the longitudinal motion had basically returned to its initial appearance.

In this study we did not expect to elicit steady‐state response at each stage of load; our goal was primarily to be able to measure the longitudinal motion during some degree of physical activity and increase in blood pressure. However, in future studies it can be interesting to study the longitudinal motion also during steady state.

In our previous study on aging (Cinthio et al., 2018) in a larger group of healthy individuals without any medication we found that LMovMax seemed to decrease at high age. In that study, the material was larger and the age‐range wider, and the decrease seemed to come at an older age than the age of the subjects in the present study, thus the finding at rest in the present study is consistent with our previous findings.

Shear strain is normally defined on a bulk medium, whereas the measurement of shear strain in this study is performed on vessel wall tissue, which is inhomogeneous. Since we cannot study all the arterial wall layers separately, due to the resolution of the ultrasound scanner, we have chosen to use a simplified model and thus in the calculations view the arterial wall as one medium. However, we have previously reported that the largest longitudinal displacement take place in or close to the demarcation between the media and the adventitia, that is, most likely at the external elastic lamina (Nilsson et al., 2010).

In conclusion, this study shows that the multiphasic bidirectional longitudinal motion of the human carotid artery undergoes marked changes during exercise already at low workload, with most evident a marked increase/emergence of an antegrade motion in early systole. The increase seems to be linked to increase in blood pressure but not to blood flow velocity or wall shear stress. The results of this study give further support to our previous notion that the longitudinal motion of the arterial wall can be of fundamental importance for vascular hemodynamics and biology, and thus, for the study of atherosclerosis and vascular diseases.

## ETHICS STATEMENT

The study was approved by the Ethics Committee, Lund University, and informed consent according to the Helsinki Declaration was obtained from all subjects.

## FUNDING INFORMATION

This study was supported by grants from the Swedish Research Council, the Medical Faculty, Lund University, the Skåne County Council's Research and Development Foundation, and Funds at Skåne University Hospital.

## CONFLICTS OF INTEREST

The authors have no conflicts of interest.
